# Supplementary material for: A Critical View of the Application of the APEX Software (Aqueous Photochemistry of Environmentally-Occurring Xenobiotics) to Predict Photoreaction Kinetics in Surface Freshwaters
Source: Molecules. 2019 Dec 18;25(1):9. doi: 10.3390/molecules25010009 (PMC7017383; doi:10.3390/molecules25010009)
Supplement: Supplementary file 1 [file molecules-25-00009-s001.zip › 3.2.4_gcc-4.4.0.zip!/3.2.4_gcc-4.4.0/3.2.4_gcc-4.4.0/doc/octave/html/interpreter/Cursor-Motion.html]

Cursor Motion - Untitled


Next: Killing and Yanking,
Up: Command Line Editing


---

#### 2.4.1 Cursor Motion

The following commands allow you to position the cursor.

`C-b`: Move back one character. `C-f`: Move forward one character. `<DEL>`: Delete the character to the left of the cursor. `C-d`: Delete the character underneath the cursor. `M-f`: Move forward a word. `M-b`: Move backward a word. `C-a`: Move to the start of the line. `C-e`: Move to the end of the line. `C-l`: Clear the screen, reprinting the current line at the top. `C-_` `C-/`: Undo the last action. You can undo all the way back to an empty line. `M-r`: Undo all changes made to this line. This is like typing the `undo' command enough times to get back to the beginning.

The above table describes the most basic possible keystrokes that you need
in order to do editing of the input line. On most terminals, you can
also use the left and right arrow keys in place of `C-f` and `C-b`
to move forward and backward.

Notice how `C-f` moves forward a character, while `M-f` moves
forward a word. It is a loose convention that control keystrokes
operate on characters while meta keystrokes operate on words.

The function `clc` will allow you to clear the screen from within
Octave programs.

— Built-in Function: **clc** ()  
— Built-in Function: **home** ()  
> Clear the terminal screen and move the cursor to the upper left corner.
